# Prior Authorization of Medication and Its Influence on Provider Behavior: Latent Class Analysis

**DOI:** 10.2196/75361

**Published:** 2025-07-29

**Authors:** Stephen Salzbrenner, Lawrence M Scheier, Fang Qiu

**Affiliations:** 1 Department of Psychiatry, University of Nebraska Medical Center, 985575 Nebraska Medical Center, Omaha, 68198, United States, 1 4025526007, 1 4025526035; 2 Lars Research Institute, Sun City, United States; 3 Department of Biostatistics, University of Nebraska Medical Center, Omaha, NE, United States

**Keywords:** prior authorization, step therapy, prescribing behavior, latent class analysis, insurance, pharmacy benefit manager

## Abstract

**Background:**

Insurance companies frequently require prior authorization (PA) for medication prescriptions to ensure quality control and safety. The added layer of scrutiny can contribute to provider dissatisfaction and has been associated with adverse patient outcomes. Health care providers have changed prescribing behaviors to avoid PA. Understanding factors contributing to this phenomenon can facilitate systemic change and better patient care.

**Objective:**

The objectives of this study are to identify unique unobserved subgroups of prescribers with similar PA-related behaviors using a finite mixture modeling approach; characterize subgroup membership by important covariates; and examine the influence of subgroup membership on 3 relevant prescribing outcomes.

**Methods:**

A cross-sectional, web-based, nationwide survey of 1173 prescribers was oversampled for psychiatry in support of developing a software-as-a-solution to facilitate PA. Latent class analysis included 12 indicators assessing the degree of PA involvement, provider-insurance communication, and the methods of obtaining or avoiding PA. Covariates included age, gender, race, provider role, specialty, number of prescribers, and patient load. Three clinical decision outcomes included prescribing medication other than initially preferred due to PA delays, avoiding newer medications due to anticipated need for PA, and modifying a diagnosis to obtain PA.

**Results:**

In total, 1147 prescribers responded with 1144 usable surveys (age, median 50.003 [range 25.00, 72.00] years; 569 (49.74%) females; 67.13% White; 44.84% psychiatrists). In total, 4 unique classes were obtained based on 12 indicators assessing PA-related activities. Classes included a high PA denial class (291 [25.15%]), a Low Volume PA (178 [15.93%]), a class denoted by Problematic Communication Issues with insurers (227 [19.96%]), and a Low Volume PA Class with Problematic Experiences (446 [38.97%]). Only 3 of the 7 covariates (age, specialty type, and patient load) provided additional means to characterize class membership. The observation that certain demographics (race and gender) and provider characteristics (specialty) may not be informative has policy implications and can inform means to improve provider-insurer communication. The largest class reporting problematic PA experiences had significantly higher mean levels for changing their prescribing and diagnostic behaviors than the remaining classes.

**Conclusions:**

Providers are not homogeneous regarding their experience with PA and insurance companies. It is, therefore, important to recognize subtle behavioral differences and find ways to accommodate the PA process to their unique needs. This will facilitate the appropriate implementation of PA by insurance companies. Providers can then avoid the need to alter medications, change diagnoses, or resist prescribing newer, effective medications that may require lengthy clinical documentation.

## Introduction

Prior authorization (PA) is used by health insurers to manage access to costly medications and ensure their safe, effective, and value-based use [1]. However, PA can negatively impact workflow, patient care, and provider satisfaction. An American Medical Association survey reported that providers spend a mean of 12 hours on PAs per week [2]. Moreover, 95% of physicians reported that PA had a somewhat or significant negative impact on clinical outcomes, including delayed access to care, treatment abandonment by patients, and serious adverse health events. Prescribers reported that 31% (310/1000) of PAs are often or always denied. 88% (880/1000) of physicians reported that PA led to higher overall utilization of health care resources, including ineffective initial treatment, additional office visits, immediate care and emergency room visits, and hospitalizations. To address these issues, 40% (400/1000) of physicians have staff who work exclusively on PA.

The 2019 ePA National Adoption Scorecard by CoverMyMeds noted that the type of medical specialty can also contribute to PA burden [3]. More in-depth qualitative studies reinforce provider burden due to extensive paperwork and inconsistent PA requirements among health plans [4,5]. Given these consequences and the burden of PA, providers, pharmacists, policy makers, and other stakeholders have supported efforts to limit, standardize, and streamline PA processes [6-11].

Although there is a body of evidence on the benefits and unintended consequences of PA, we could only find 1 published study that examined the effect of PA on providers’ clinical decision-making. This involved a survey of 326 psychiatrists in which a majority reported at least occasionally using tactics including diagnosis modification or falsification of previous medication trials to obtain PA [12]. An additional two-thirds refrained at least occasionally from prescribing preferred medications due to an actual PA requirement or expectation of one. This gap in the literature prompted us to conduct a nationwide survey of ~1200 prescribers representing all but 7 states, which examined clinical practices such as modifying diagnoses, avoiding evidence-based medications, or avoiding prescribing newer medications in relation to various PA burdens and clinical factors [13]. The results of this study as well as survey data from various sources [5,10,11,13] reveal that not all providers feel the same about the PA process, nor do they modify their clinical practices in the same way to avoid problems with PA. When it comes to directly interfacing with health insurers over the issue of PA, one size does not fit all, suggesting there may be manifold provider experiences. Subgroups of providers may exist differentiated based on the tenor of their interactions with PA, owing to differences in the volume of PA, patient load, the quality of provider-insurer interactions, and insurers’ demands to provide support for a particular prescription or course of treatment. To our knowledge, no study has yet examined subgroups of providers who have unique experiences revolving around PA nor determined whether these subgroups differ in prescribing and diagnostic behaviors.

In the present study, we rely on latent class analysis (LCA), a mixture model approach to determine whether providers form qualitatively distinct subgroups (classes) based on their day-to-day interactions with insurers over PA. The classes differ qualitatively rather than quantitatively because the focus is on “response patterns,” not distributional behaviors like a measure of central tendency (eg, mean) would represent. Mixture models are part of a broad class of person-centered analytic techniques that examine relations between people rather than between variables, as with a variable-centered approach (ie, correlation or regression). It is considered a categorical analogue to factor analysis where the underlying latent factor is categorical (and has a multinomial distribution rather than a continuous distribution), and the indicators for the categorical latent factor are themselves also categorical. The different levels of the categorical latent factor correspond to unique (mutually exclusive and exhaustive) “subgroups” that share behavioral similarity [14,15]. To illustrate, if there are 2 survey questions, each with response formats of “yes” and “no,” there would be 2^2^ possible response patterns (YY, YN, NY, and NN). When there are 8 survey questions, there are 2^8^ or 256 possible response patterns, which makes it a bit trickier for the naked eye to detect the composition of unique classes. Some type of assignment process is needed that can accurately predict the different response patterns based on the empirical data. This is where LCA can discern meaningful patterns in the data based on probability theorems using a multiway contingency table. Individuals are assigned to their respective class or subgroup based on estimated posterior probabilities using the joint marginal distributions of survey items. There is a margin of error in the assignment process as class membership cannot be perfectly predicted for any individual (perfect prediction of class membership would create a nominal observed variable like gender or race). Once mutually exclusive subgroups were obtained, we addressed whether they can be further characterized by demographics and other relevant covariates. Following this procedure, we modeled the relationships between class membership and 3 measures of clinical decision-making that reflect provider behaviors associated with PA experience. As explained below, this analysis provides insight into clinicians’ diagnostic and prescribing behavior and whether it differs based on their unique class membership.

## Methods

### Recruitment

A 58-item survey was administered in October 2020 using the Qualtrics platform. Invitation emails with a unique hyperlink were sent to ~100,000 licensed providers with emails drawn from a curated, nationwide list. The study oversampled psychiatrists to address PA in mental health care settings. The survey took ~10 minutes to complete ($\overset{-}{X}$=3.73). A handful of surveys had to be discarded because providers started the web-based survey but failed to produce sufficient usable data (97.8% usable with 98.11% survey completion). The response rate for the survey was 1.2%. Additional details of survey administration and sampling procedures can be found in the study by Salzbrenner et al [13,16].

### Measures

We used a total of 12 latent class indicators to model subgroup membership. These included number of PAs completed in a week, number of hours spent on PA, length of time waiting for PA decisions from health plans (past week), length of time to complete PAs, percentage of medication requests approved upon appeal, challenges associated with identifying appropriate step therapy requirements prior to prescribing medication, needing to send additional clinical documentation, not being notified by the insurer of a medication approval, not being notified of a medication denial, and being denied PA because the request was missing specific adverse effects of past medication, because of dosing issues, and because of formulation issues. Collectively, these 12 measures capture the providers’ degree of engagement with PA, challenges associated with PA (ie, barriers and obstacles), and provider-insurer communication issues revolving around PA.

All indicators were dichotomized to 0/1, where “1” indicates heavy involvement in PA and numerous challenges. Support for dichotomization is provided when the goal is to acknowledge that a provider’s experience with PA has occurred (yes or no), rather than modeling distributional behavior with central moments [17,18]. Covariates in the model include provider characteristics (age, race, and gender), type of provider (DO/MD vs nurse practitioner [NP]/physician assistant), provider subspecialty (psychiatrist vs all others), and practice characteristics (active patient load and the number of providers that can prescribe medications). Three continuous measures were modeled as “distal outcomes.” These included, “In what percentage of cases do you prescribe a different medication than initially planned due to prior authorization delays?” (ranging from 1 “less than 10%” to 5 “>50%”); “How often do you avoid prescribing newer medications due to anticipated difficulties with prior authorization, even if you feel patients meet evidence-based guidelines for their use?” (ranging from 1 “very rarely” to 5 “extremely often”); and “How often have you modified a diagnosis to obtain a prior authorization?” (ranging from 1 “rarely” to 5 “extremely often”).

The LCA analyses were conducted using Mplus statistical software [19]. Imputation procedures to correct missing data for the covariates were conducted using the MICE procedure in R [20,21]. This is a fully conditioned imputation using predictive mean matching, which considers the distributional characteristics of each missing variable in a multivariate framework [22]. We used 20 imputations, which is sufficient to obtain unbiased parameter estimates [23].

We first tested LCA models with 2-8 classes. Selection of the best fitting model was based on the Akaike information criterion [24], Bayesian information criterion [25], entropy [26], and the log-likelihood statistical fit index (LL). These statistics provide a means to gauge whether a model with *k*–1 classes vs *k* classes is superior in fit. As more classes are extracted, there should be a modicum of shrinkage in the information criteria. The LL statistic reflects the likelihood of observing the empirical data given the set of parameter estimates (the logarithm of the LL is used so that higher values closer to 0 indicate better fit). Entropy (ranging from 0 to 1) is a standardized measure that reflects the chaos of a model, with values closer to 1 denoting better classification certainty. Conceptually, we looked for evidence of clear class separation with distinct response patterns for the different classes (ie, class enumeration) [27]. We also want to avoid small or sparse cells (<5%) that may not generalize or replicate [28].

Following derivation of the class structure, we covariate adjusted the model using the R3STEP procedure available in the Mplus software program [29] (see Supplement 1 in Multimedia Appendix 1 for an explanation of how this procedure works). We then modeled relations between the class structure and 3 distal outcomes using the Bolck, Croon, and Hagenaars (BCH) procedure available in the Mplus statistical program [30] (see Supplement 2 in Multimedia Appendix 1 for more about the BCH procedure). Mixture model and subsequent multinomial logistic model analyses were conducted with weights to adjust for non-response. In total, 5 auxiliary variables were used to compute weights including sex, age, practitioner role (MD/DO vs NP and physician assistant), and specialty type (available for both respondents and nonrespondents). Weights were obtained using iterative logistic regression predicting presence in the sample vs the population (delimiting sample data from the population file to avoid overstating presence). This was done to approximate population values and implemented using propensity weighting strategies to make the sample distributions match to the total sample (ie, the known population distribution). This generates a response probability for each auxiliary measure. A bias statistic was estimated as the difference in parameters between the expected value (based on the population of providers) and the sample value (regression coefficients and SEs). The results were virtually identical and thus for ease of interpretation we only report unweighted results in this paper.

### Statistical Analysis

We used a Monte Carlo simulation to estimate power with the LCA analyses [31]. With a finite mixture model the question of power revolves around having sufficient sample to extract the right number of classes [27]. Computing power for a mixture model is not as straightforward as with regression or factor analysis. This is because traditional power with precise parameter estimates cannot be used given there are boundary conditions for the parameter estimates (item response probabilities [IRPs] and latent class prevalence are between 0 and 1 and the number of classes is indeterminate). Sample size estimation and power considerations with mixture models can be determined using a Monte Carlo simulation. We specified a model with 5 covariates and up to 5 classes, varying the thresholds (logits) for class composition. The simulation model used maximum likelihood estimation using an expectation maximization algorithm with 10,000 replications (the sample size for the study with a goal of gaining stability in the parameter values) and averaging parameter values across these samples.

The study has sufficient power (≥.80) to obtain adequate coverage (the proportion of replications for which the 95% CI contains the true parameter value), with low levels of parameter bias (computed as the simulated parameter value averaged over the replications–population parameter value/population parameter value and not exceeding 10% for parameter bias and 5% for standard error bias). In all cases power is interpreted as the proportion of replications in which the null hypothesis stating the parameter is zero can be rejected at the .05 level of significance (ie, the probability of rejecting the null when it is false).

### Ethical Considerations

All procedures performed in studies involving human participants were in accordance with the ethical standards of the institutional or national research committee and with the 1964 Declaration of Helsinki and its later amendments or comparable ethical standards. Informed consent was obtained from all individual participants included in the study. The study received Institutional Review Board approval from the University of Nebraska Medical Center (IRB # 00000672 Protocol # 423‐19-EP). The providers received a US $10 gift card upon completion.

## Results

### Participant Characteristics

The sample was 48.9% (574/1173) female with a mean age of 50.5 (SD 12.9) years. A majority were MD/DO providers (76%) with a smaller percentage NPs (14%) or physician assistants (10%). A majority of the sample was White (67%), followed by Asian (18%) and 4% identified as non-White Hispanic. The largest proportion of respondents were in Psychiatry per study design (44.9%), followed by Internal Medicine (18%), Dermatology (13.1%), Gastroenterology (8.0%), Neurology (5.9%), Oncology (5.8%), and Rheumatology (4.4%).

Table S1 in Multimedia Appendix 2 contains additional sample information, including comparisons based on gender, race, and practice subspecialty. The largest share of providers (44%) worked in practices with less than 5 prescribers, with 23.5% having between 5 and 10, and another 18% having 20 or more (the remaining percentages were much smaller). Active patient load varied considerably, with the largest number (48%) having over 200 patients, while remaining providers were equally split among smaller practices (<25, 11%; 25‐50, 13%; 51‐100, 13%; 101‐200, 14%). The size of practices varied considerably, with the majority (67%) having less than 5 advanced practice providers, 18% having between 5 and 10, and the rest being much smaller practices ranging from 2% having 16%‐20% to 6.5% having 20 or more providers.

### LCA Results

Table S2 in Multimedia Appendix 3 contains the model fit indices corresponding to the 2‐8 class LCA models. Upon careful inspection, the 4-class model provided the best fit, noted by shrinkage in the Akaike information criterion, Bayesian information criterion, and LL statistic with the progressive extraction of classes. Relative entropy was less helpful in determining which model to choose, as the values fluctuated up and down with the extraction of additional classes. We further inspected the pattern of IRPs for all the models, looking for any distinguishing features of class membership and the latent class prevalence for the different classes within each model. The goal is to select the most parsimonious model that most efficiently captures the underlying behaviors of the sample participants while simultaneously obtaining the best class enumeration based on the unique response patterns.

**Figure 1.**
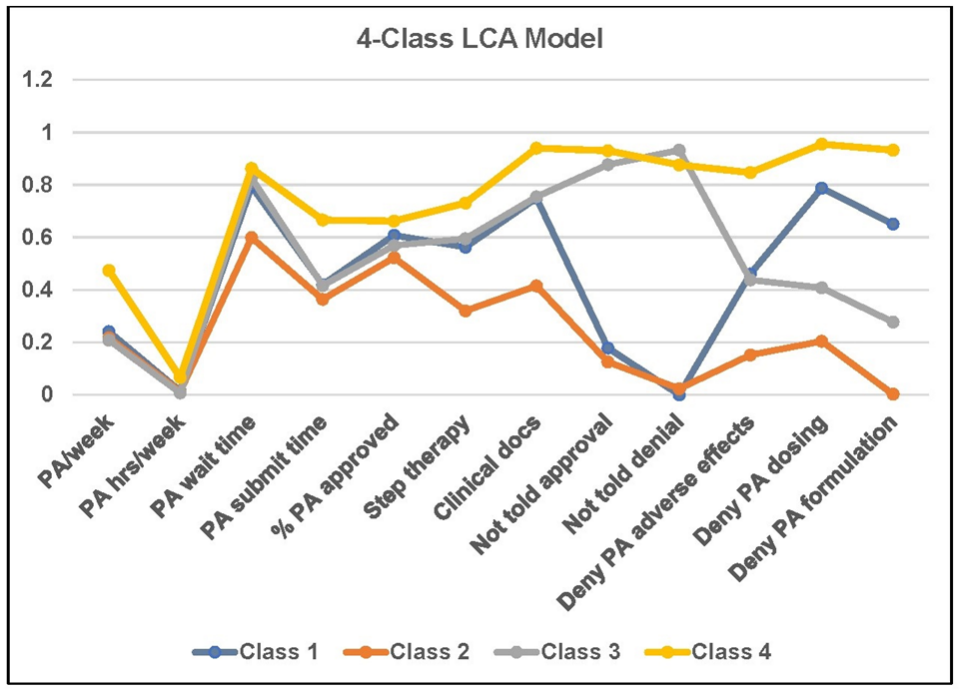
4 Class latent class analysis (LCA) model. PA: prior authorization.

Table 1 shows the IRPs for the 4-class model and should be read in conjunction with Figure 1, which graphically portrays the IRPs. The IRPs indicate the probability of endorsing an item conditional on class membership. Class 1 (25.44%) consisted of providers who endorsed lengthy waiting times for PA decisions from health plans (ρ=.794), a high percentage of denied medications approved upon appeal (ρ=.608), the need to send additional documentation (ρ=.750), and PA denial because of dosing (ρ=.788) or formulation issues (ρ=.651). Considering this, we labeled this class “High Denial PA.” Class 2 (15.58%) was distinguished because they only endorsed 1 item close to the .6 threshold for excessive waiting time for PA (ρ=.599). Given that members of this class did not endorse any other items above the .6 threshold, we labeled it “Low Volume PA*.*” Class 3 (19.91%) had 4 items above the critical .6 threshold, including long average wait for PA (ρ=.826), challenges with step therapy (ρ=.595), which is reasonably close to the .6 threshold, having to send additional clinical documentation (ρ=.754), not being notified of medication approval (ρ=.877), and not being notified of medication denial (ρ=.932). We labeled this class “Problematic Communication Issues.” Class 4 (39.06%) was distinguished by the fact that its members endorsed almost all indicators except for the number of PAs completed in a week (ρ=.474) and hours spent personally on PA (ρ=.066). In the case of the latter parameter, it means that members of this class did not spend a lot of hours working with PA. The remaining indicators were highly endorsed (avg. ρ=.840) and as a result, we labeled this class “Problematic PA Experiences.” It is notable that class 4 endorsed significant workflow burden despite the fact that members of this class did not spend significant time per week on PA. This could be reflective of administrative delegation.

**Table 1.** Item response probabilities for the 4-class model.

Item	Latent class			
	1^a^	2^b^	3^c^	4^d^
Class prevalence	25.44%	15.58%	19.91%	39.06%
# prior authorizations completed in a week	0.241	0.217	0.209	0.474
# hours personally spent on prior authorization (PA) per week	0.014	0.012	0.007	0.066
Average wait for PA decision from health plan	0.794^e^	0.599	0.826^e^	0.863^e^
Average time PA completion or submission	0.42	0.364	0.416	0.666^e^
Percentage of denied medication requests approved on appeal	0.608^e^	0.522	0.569	0.662^e^
Challenge to identify appropriate step therapy requirements	0.562	0.32	0.595	0.731^e^
Necessary to send additional clinical documentation for medication	0.75^e^	0.415	0.754^e^	0.94^e^
Not notified of medication approval	0.178	0.125	0.877^e^	0.93^e^
You are not notified of medication denial	0.000	0.023	0.932	0.876^e^
Deny PA request missing adverse effects of past medications	0.461	0.152	0.438	0.847^e^
Deny PA request because of dosing issues	0.788^e^	0.204	0.407	0.955^e^
Deny PA request because of formulation issues	0.651^e^	0.002	0.277	0.932^e^

a Class labels: Class 1=High Denial PA.

b Class 2=Low Volume PA.

c Class 3=Problematic Communication Issue.

d Class 4=Problematic PA Experiences.

e The numbers represent probabilities exceeding .600 (ie, 60% endorsement of some type of issue with PA).

Table 2 contains the results of the covariate-adjusted models including the univariate models (upper portion) and multivariate (lower portion) multinomial logistic regression models. The univariate model determines whether a covariate is significantly related to class membership and can be used to detect evidence of suppression in the multivariate model. Only 3 of the 7 covariates, including age, specialty, and patient load (in both the univariate and multivariate models), were significantly related to class membership. In the adjusted models, older providers were 18% more likely to be members of the High Denial PA class compared to the Problematic PA Experiences reference class. Members of the Low Volume PA class were 2% more likely to be older compared to the reference class. Members of the High Denial PA class were over 2 times more likely to be psychiatrists compared to the reference class. Members of the Low Volume PA class as well as those in the Problematic Communication Issues class were less likely to have a high patient load (ORs=0.49 and 0.54, respectively) compared to the reference class 4.

**Table 2.** Multinomial logistic regression predicting class membership.

	Latent class^a^						
	1^b^		2^c^		3^d^		4^e^
	OR (95% CI)	*P* value	OR (95% CI)	*P* value	OR (95% CI)	*P* value	
Prevalence	25.44%		15.58%		19.91%		39.06%
Unadjusted OR^f^							
Age	1.023 (1.007, 1.039)	.005	1.02 (1.001, 1.039)	.038	0.998 (0.979, 1.017)	.797	Ref.
Sex^g^	0.812 (0.557, 1.183)	.278	0.735 (0.474, 1.139)	.168	0.903 (0.582, 1.4)	.648	Ref.
White^h^	0.995 (0.655, 1.51)	.98	0.818 (0.503, 1.331)	.418	1.116 (0.677, 1.838)	.668	Ref.
Specialty^i^	2.252 (1.532, 3.311)	<.001	1.132 (0.714, 1.793)	.598	1.28 (0.824, 1.988)	.271	Ref.
Provider role^j^	1.421 (0.913, 2.211)	.119	1.627 (0.961, 2.754)	.07	1.22 (0.742, 2.005)	.433	Ref.
Prov_Rx^k^	0.795 (0.548, 1.152)	.226	0.738 (0.481, 1.131)	.163	1.158 (0.743, 1.805)	.518	Ref.
Pt_load^l^	0.894 (0.549, 1.455)	.652	0.51 (0.307, 0.847)	.009	0.545 (0.33, 0.901)	.018	Ref.
Adjusted OR^m^							
Age	1.018 (1.001, 1.036)	.039	1.021 (1, 1.042)	.045	0.997 (0.977, 1.018)	.797	Ref.
Sex	0.972 (0.639, 1.478)	.894	0.889 (0.543, 1.456)	.641	0.952 (0.582, 1.558)	.846	Ref.
White	0.873 (0.56, 1.36)	.549	0.755 (0.447, 1.275)	.293	1.213 (0.697, 2.11)	.495	Ref.
Specialty	2.069 (1.392, 3.076)	<.001	0.943 (0.577, 1.542)	.816	1.243 (0.77, 2.006)	.373	Ref.
Provider role	1.167 (0.709, 1.923)	.543	1.531 (0.847, 2.768)	.159	1.181 (0.641, 2.176)	.594	Ref.
Prov_Rx	0.97 (0.654, 1.436)	.877	0.782 (0.495, 1.235)	.291	1.216 (0.745, 1.983)	.434	Ref.
Pt_load	0.909 (0.546, 1.513)	.713	0.49 (0.292, 0.824)	.007	0.537 (0.324, 0.891)	.016	Ref.

a Based on estimated posterior probabilities [19].

b Class labels: Class 1=High Denial.

c Class 2=Low Volume PA.

d Class 3=Problematic Communication Issues.

e Class 4=Problematic PA Experiences.

f Covariates entered one at a time.

g Reference class for each covariate is 0: sex (M=0, F=1).

h White (Other=0, White=1).

i Specialty (Other=0, Psychiatry=1),

j Provider role (Other=0, DO/MD=1).

k Providers who write Rx (Other=0, # of providers ≥5 =1).

l Patient load (Other=0, >50 patients=1).

m Covariates entered as a block [19].

### Distal Outcomes

With the BCH procedure, individuals are assigned to their most likely class (see Multimedia Appendix 1 for more on this procedure), creating a nominal variable that can be used for subsequent variable-centered analyses. Tables3  4 show the results of this procedure, including the estimated means for each class (Table 3) and the pairwise comparisons of intercepts between classes (Table 4). The stepwise modeling procedure included contrasting intercepts when only class membership is covariate-adjusted (controlling for unique characteristics of individuals within-class) and then covariate-adjusting the distal outcomes. These adjustments avoid spurious findings when characteristics associated with class membership influence the outcome indirectly or directly influence the outcomes. Of the 18 pairwise comparisons, 14 were significant and only 3 would be eliminated with a Bonferroni-type adjustment for multiple comparisons. A positive mean difference indicates the first class had a larger mean. Overall, the Problematic PA Experiences Class 4 had significantly higher means for altering clinical decision-making because of PA issues compared to the remaining 3 classes. This held for all 3 distal outcomes (the mean differences were all negative). Class 2 (Low Volume PA), on the other hand, which was characterized by the lowest endorsement of PA problems, had much lower means than the remaining classes for all 3 clinical decision-making outcomes.

**Table 3.** The estimated distal outcomes per class^a^.

Outcome	Latent class	Mean	SE
Q14^b^	C1^c^	3.274	0.278
	C2^d^	2.452	0.275
	C3^e^	3.36	0.27
	C4^f^	3.712	0.257
Q15^g^	C1	3.102	0.231
	C2	2.602	0.249
	C3	3.22	0.223
	C4	3.527	0.218
Q16^h^	C1	2.045	0.201
	C2	1.835	0.2
	C3	2.173	0.197
	C4	2.538	0.191

a All BCH models controlled for covariates.

b LABELS: Q14=Prescribe a different medication due to prior authorization (PA) delays.

c Class 1=High Denial PA.

d Class 2=Low Volume PA.

e Class 3=Problematic Communication Issues.

f Class 4=Problematic PA Experiences.

g Q15=Avoid prescribing newer medication due to PA.

h Q16=Modifieda diagnosis to obtain PA.

**Table 4.** Pairwise comparisons for the latent class analysis (LCA) model with distal outcomes^a^.

Outcome	Latent class	Mean difference	SE	*P* value
Q14^b^	1^c^ vs 2^d^	0.821	0.184	<.001
	1 vs 3^e^	−0.086	0.16	.591
	1 vs 4^f^	−0.439	0.13	.001
	2 vs 3	−0.907	0.182	<.001
	2 vs 4	−1.26	0.146	<.001
	3 vs 4	−0.353	0.15	.019
Q15^g^	1 vs 2	0.5	0.161	.002
	1 vs 3	−0.118	0.13	.362
	1 vs 4	−0.426	0.107	<.001
	2 vs 3	−0.619	0.165	<.001
	2 vs 4	−0.926	0.133	<.001
	3 vs 4	−0.307	0.123	.013
Q16^h^	1 vs 2	0.211	0.112	.06
	1 vs 3	−0.127	0.107	.232
	1 vs 4	−0.493	0.093	<.001
	2 vs 3	−0.338	0.112	.003
	2 vs 4	−0.704	0.094	<.001
	3 vs 4	−0.366	0.108	.001

a All models controlled for covariates.

b LABELS Q14=Prescribe a different medication due to prior authorization (PA) delays.

c Class 1=High Denial PA.

d Class 2=Low Volume PA.

e Class 3=Problematic Communication Issues.

f Class 4=Problematic PA Experiences.

g Q15=Avoid prescribing newer medication due to PA.

h Q16=Modified a diagnosis to obtain PA.

## Discussion

### Principal Findings

We identified 4 distinct classes based on PA-related insurance interactions, PA volume, and various challenges they confront as part of the PA process. These qualitative distinctions have not been noted in the literature, which has focused on descriptively showing the prevalence of providers who encounter problems. This glosses over the fact that not all providers share the same sentiment or have identical PA experiences. Understanding the nature of these experiences and the composition of different subgroups may foster corrective actions to improve efficiency while decreasing provider burden. We also examined whether the unique classes are different in their respective clinical decisions regarding prescribing and diagnosis. The latter issue gets at the heart of how PA affects providers and the effect of PA on medical practice.

The largest Class 4 (Problematic PA Experiences) encountered the most problems in every facet of the PA process. Although they had low volumes of PA and spent very few hours engaged in PA, they reported waiting extensively for PA decisions, experiencing frequent denials, challenges with step therapy, responding to requests for additional clinical documentation, not being notified of approvals or denials, and encountering denials because of missing adverse medication effects, dosing issues, or formulation issues. In contrast, the smallest class endorsed waiting for PA decisions as the sole challenge faced. The 2 remaining classes endorsed a few problems but in no consistent or definable pattern that could distinguish their PA experiences.

All 4 classes were distinguished by minimal endorsement of 2 questions: how many PAs are completed in a week and the number of hours personally spent on PA. These patterns may indicate either that the medical providers have dedicated staff addressing these problems or that providers represent practices with relatively low volumes of PAs. In the latter case, they still encountered problems, as evidenced by the way they endorsed the other PA-related survey questions. Practically speaking, the sample consisted of a fair representation of different specialties, favoring a larger share of psychiatrists by design (45%) but including specialties like Internal Medicine and Dermatology that routinely have heavy PA exposure.

Modeling covariates helps to further characterize class membership. Of the 7 covariates, age, specialty, and patient load were the most prominent measures to distinguish class membership. Members of Class 1 (High Denial PA) were older than the reference Class 4 (Problematic PA Experiences) and more likely to be psychiatrists. Members of Class 2 (Low Volume PA) were also older than the reference class, suggesting that older providers did not see PA as problematic compared to the members belonging to the Problematic PA Experiences reference class. Patient load was relatively low for members of the Low Volume PA class and likewise Class 3 (Problematic Communication Issues) compared to the reference class. Taken together, providers in the Problematic PA Experiences class were younger and had higher patient loads compared to the other classes, suggesting that large practices with younger providers experience more significant issues with PA and may want to see system-wide changes to alleviate the burdens of PA.

This study also showed that there is a significant relationship between whether providers encounter difficult challenges with PA and 3 measures of their clinical decision-making. This is a strong indication that the burdensome experiences brought about through the PA process have the ability to change medical practice by altering the treatment decisions made by providers. This raises the potential that the actions that providers take to avoid PA-related burdens could have significant downstream implications for patient safety, including undesired clinical outcomes and threats to public health. We only examined the relationship between class membership and clinical decision-making at a high level of analysis. Future studies may want to break this down and examine further what contributes to the changes in clinical decision-making and whether this can be rectified in some fashion. This type of more detailed analysis could be quite informative and influential regarding health policy and practice.

### Limitations

There are several limitations worth noting. First, the data are cross-sectional, providing only a glimpse of provider behaviors at one point in time. Longitudinal data would be required to infer some causal sequence relating, for example, provider-insurer interactions and determine if these behaviors and sentiments are stable or change progressively (for better or worse). This could entail a repeated measures design that samples provider-insurer interactions on numerous occasions and develops a model that includes change in provider and patient behaviors (ie, clinical outcomes). Second, although we sampled more than one specialty, there were several that were not included. Casting a wider net around different practice specialties might shed light on the extent of provider dissatisfaction and whether class structure is consistent across specialties. Included would be Oncology, Gastroenterology, Cardiology, and Nephrology since those specialists write a large volume of specialty prescriptions. Extending the study to include more practice types that differ by composition would also lend credence to how pervasive PA dissatisfaction is and whether it is volume or specialty dependent. Third, while dichotomization of the 12 class indicators was necessary from an analytic point of view, some of the items (eg, wait times, documentation burden) may contain significant gradations that would provide more information and richer class distinction. Therefore, the dichotomization of LCA indicators could obscure meaningful inter-class differences. Finally, the low response rate of 1.2% increases the risk of selection bias; specifically, those who are dissatisfied with PA or have issues revolving around PA may be overrepresented.

### Comparison With Prior Work

Past studies have identified provider issues with PA; however, they have treated providers as a single undifferentiated population. This assumes providers will have the same reactions and results when interacting with insurance companies over PA. Moreover, the nature of relations between PA activities and provider clinical outcomes, eg, dissatisfaction with PA procedures, has been examined only at the bivariate level of analysis. This limits what we know about PA and provider behaviors to a very small slice of experiences that are examined in an isolated manner. In the present study, the inclusion of 12 indicators capturing a more holistic set of experiences can shed light on systemic factors that affect provider behaviors.

### Conclusions

This study adds important insight into the effect of PA on providers’ experience. Factors such as age and patient load significantly influence the provider experience, as well as prescribing behaviors, which could lead to disparate health outcomes. Recognizing unique provider experiences can help facilitate optimization of patient care while decreasing provider burden.

Clinically, this study reveals a concerning trend that could have dangerous implications for patients. First, the study demonstrated that providers occasionally modify diagnoses in charts in order to avoid insurance denial or the need for PA. This means, in practical terms, that a patient with bipolar disorder may be diagnosed with “major depressive disorder” in order to get effective medication authorized if insurance only authorizes the medication for treatment of unipolar depression. The downstream implications of this can be quite pronounced, affecting public health measures that rely on analysis of this clinical data. For instance, public health officials might use the clinical data to promote health policy to address medical conditions that are not as prevalent as the records may show. Conversely, this could take important resources away from conditions that are underreported by health care providers.

## Supplementary material

10.2196/75361 Multimedia Appendix 1 Supplemental materials.

10.2196/75361 Multimedia Appendix 2 Supplemental eTable 1.

10.2196/75361 Multimedia Appendix 3 Supplemental eTable 2.

## Abbreviations

BCH: Bolck, Croon, and Hagenaars
IRP: item response probabilities
LCA: latent class analysis
LL: log-likelihood statistical fit index
NP: nurse practitioner
PA: prior authorization
