# Supplementary material for: Prior Authorization of Medication and Its Influence on Provider Behavior: Latent Class Analysis
Source: J Med Internet Res. 2025 Jul 29;27:e75361. doi: 10.2196/75361 (PMC12306842; doi:10.2196/75361)
Supplement: Multimedia Appendix 1 [file jmir-v27-e75361-s001.docx]

**Supplemental Materials**

**#1: The R3STEP Procedure**

The R3STEP procedure is one of several “*classify-analyze*” approaches to model the effect of explanatory variables and keep them structurally independent of the measurement model [1,2]. It is a flexible stepwise procedure that estimates an unconditioned LCA model to compute the conditional probabilities for modal class assignment by producing a parameter representing the average classification error. Individuals are assigned to their most likely class based on the latent class posterior probability distribution. Then with the model measurement parameters fixed (i.e., thresholds expressed as logits), and accounting for measurement error in the class assignment process, the final model is conditioned by the covariates, adjusted for uncertainty in misclassification.

**#2: The BCH Procedure**

The BCH procedure has the goal of avoiding the covariates affecting the measurement model (assigning class membership) in the same way as the R3STEP procedure. Only, in the case of distal outcomes, we relied on the BCH method available in the Mplus software [3]. The BCH procedure uses a “weighted multiple group analysis” where each group becomes one of the latent categories for the LCA model and the groups are known (observed) and modeled as a multinominal fixed variable.^4^ The BCH weights (the inverse of the matrix of classification errors) represent the measurement or classification error based on posterior probabilities and treated as a regression coefficient in the model that arises because there is uncertainty in class assignment (i.e., error probabilities). This locks in cases to their respective class and they can be compared using traditional regression or other variable-centered methods (i.e., ANOVA or t‑tests). In the case of distal outcomes, post-hoc pairwise comparisons between classes is used to statistically contrast intercepts for the three clinical decision outcomes. Significant differences are based on a critical z-ratio with the parameter divided by its respective standard error.

**References**

1. Bray BC, Lanza ST, Tan X. Eliminating bias in classify-analyze approaches for latent class analysis. *Struct. Equ. Mod.* 2015;22(1):1-11. [doi:10.1080/10705511.2014.935265](https://doi.org/10.1080/10705511.2014.935265)
2. Vermunt, J. K. (2010). Latent class modeling with covariates: Two improved three-step approaches. *Pol. Anal.* 2020;18(4):450-469, 2010. doi:[10.1093/pan/mpq025](https://doi.org/10.1093/pan/mpq025)
3. Asparouhov T, Muthén BO. Auxiliary variables in mixture modeling: Using the BCH method in Mplus to estimate a distal outcome model and an arbitrary secondary model. *Mplus Web Notes. 2014b;*21(2):1-22.
4. Bakk Z, Vermunt JK. Robustness of stepwise latent class modeling with continuous distal outcomes. *Struct. Equ. Mod.* 2016;23(1):20-31. doi:[10.1080/10705511.2014.955104](https://doi.org/10.1080/10705511.2014.955104)
