# Supplementary material for: Prior Authorization of Medication and Its Influence on Provider Behavior: Latent Class Analysis
Source: J Med Internet Res. 2025 Jul 29;27:e75361. doi: 10.2196/75361 (PMC12306842; doi:10.2196/75361)
Supplement: Multimedia Appendix 2 [file jmir-v27-e75361-s002.docx]

Table S1. Sample characteristics and demographic comparisons

|  |  | Gender* | | | | Race* | | | | Specialty | | | |
| --- | --- | --- | --- | --- | --- | --- | --- | --- | --- | --- | --- | --- | --- |
|  | Total (N=1144) | Men (N=507) | Women (N=569) | P-value | Other (N=282) | | White (N=768) | P-value | Other (N=631) | | Psychiatry (N=513) | P-value |  |
|  |  |  |  |  |  | |  |  |  | |  |  |  |
| **Age** |  |  |  | <.001^a^ |  | |  | <.001^a^ |  | |  | <.001^a^ |  |
| N (Missing) | 990 (154) | 459 (48) | 528 (41) |  | 255 (27) | | 718 (50) |  | 543 (88) | | 447 (66) |  |  |
| Median (Range) | 50.00 (25.00, 72.00) | 52.00 (25.00, 72.00) | 47.00 (25.00, 72.00) |  | 43.00 (25.00, 72.00) | | 52.00 (28.00, 72.00) |  | 47.00 (25.00, 72.00) | | 52.00 (25.00, 72.00) |  |  |
| **Sex** n (%) |  |  |  | -- |  | |  | 0.052^2^ |  | |  | 0.015^b^ |  |
| 0 (Male) | 507 (44.32%) | -- | -- |  | 147 (52.31%) | | 348 (45.55%) |  | 262 (43.81%) | | 245 (51.26%) |  |  |
| 1 (Female) | 569 (49.74%) | -- | -- |  | 134 (47.69%) | | 416 (54.45%) |  | 336 (56.19%) | | 233 (48.74%) |  |  |
| Missing | 68 (5.94%) | -- | -- |  | 1 | | 4 |  | 33 | | 35 |  |  |
| **Race (White)**, n (%) |  |  |  | 0.052^b^ |  | |  | -- |  | |  | 0.047^b^ |  |
| 0 (Other) | 282 (24.65%) | 147 (29.70%) | 134 (24.36%) |  | -- | | -- |  | 171 (29.28%) | | 111 (23.82%) |  |  |
| 1 (White) | 768 (67.13%) | 348 (70.30%) | 416 (75.64%) |  | -- | | -- |  | 413 (70.72%) | | 355 (76.18%) |  |  |
| Missing | 94 (8.22%) | 12 | 19 |  | -- | | -- |  | 47 | | 47 |  |  |
| **Specialty**, n (%) |  |  |  | 0.015^b^ |  | |  | 0.047^b^ |  | |  | -- |  |
| 0 (Other) | 631 (55.16%) | 262 (51.68%) | 336 (59.05%) |  | 171 (60.64%) | | 413 (53.78%) |  | -- | | -- |  |  |
| 1 (Psychiatry) | 513 (44.84%) | 245 (48.32%) | 233 (40.95%) |  | 111 (39.36%) | | 355 (46.22%) |  | -- | | -- |  |  |
| **Provider Role**, n (%) |  |  |  | <.001^b^ |  | |  | <.001^b^ |  | |  | <.001^b^ |  |
| 0 (Other) | 274 (23.95%) | 35 (6.90%) | 225 (39.54%) |  | 32 (11.35%) | | 225 (29.30%) |  | 205 (32.49%) | | 69 (13.45%) |  |  |
| 1 (DO/MD) | 870 (76.05%) | 472 (93.10%) | 344 (60.46%) |  | 250 (88.65%) | | 543 (70.70%) |  | 426 (67.51%) | | 444 (86.55%) |  |  |
| **Providers write Rx**, n (%) |  |  |  | 0.969^b^ |  | |  | <.001^b^ |  | |  | <.001^b^ |  |
| 0 (<5) | 505 (44.14%) | 218 (43.17%) | 245 (43.29%) |  | 85 (30.58%) | | 362 (47.20%) |  | 248 (39.55%) | | 257 (50.20%) |  |  |
| 1 (>=5) | 634 (55.42%) | 287 (56.83%) | 321 (56.71%) |  | 193 (69.42%) | | 405 (52.80%) |  | 379 (60.45%) | | 255 (49.80%) |  |  |
| Missing | 5 (0.44%) | 2 | 3 |  | 4 | | 1 |  | 4 | | 1 |  |  |
| **Patient Load** n (%) |  |  |  | 0.522^b^ |  | |  | 0.260^b^ |  | |  | 0.583^b^ |  |
| 0 (<=50) | 274 (23.95%) | 113 (22.42%) | 137 (24.08%) |  | 72 (25.53%) | | 170 (22.22%) |  | 147 (23.41%) | | 127 (24.80%) |  |  |
| 1 (>50) | 866 (75.70%) | 391 (77.58%) | 432 (75.92%) |  | 210 (74.47%) | | 595 (77.78%) |  | 481 (76.59%) | | 385 (75.20%) |  |  |
| Missing | 4 (0.35%) | 3 | 0 |  | 0 | | 3 |  | 3 | | 1 |  |  |
| *Notes*: *68 cases had missing values for gender, and 94 had missing values for race. ^a^Wilcoxon rank-sum test p-value. ^b^Chi-Square test p-value. | | | | | | | | | | | | |  |
