# Supplementary material for: Prior Authorization of Medication and Its Influence on Provider Behavior: Latent Class Analysis
Source: J Med Internet Res. 2025 Jul 29;27:e75361. doi: 10.2196/75361 (PMC12306842; doi:10.2196/75361)
Supplement: Multimedia Appendix 3 [file jmir-v27-e75361-s003.docx]

Table S2. Model Fit Statistics for Latent Class Analyses

| **Classes** | **LL (Deviance)** | **No. of free Parameters** | **AIC** | **BIC** | **Relative Entropy** |
| --- | --- | --- | --- | --- | --- |
|  |  |  |  |  |  |
| 2 | -7666.122 | 25 | 15382.243 | 15508.301 | 0.749 |
| 3 | -7534.454 | 38 | 15144.909 | 15336.516 | 0.788 |
| 4 | -7467.820 | 51 | 15037.639 | 15294.796 | 0.760 |
| 5 | -7430.566 | 64 | 14989.131 | 15311.837 | 0.759 |
| 6 | -7401.131 | 77 | 14956.262 | 15344.518 | 0.745 |
| 7 | -7377.818 | 90 | 14935.637 | 15389.442 | 0.763 |
| 8 | -7361.324 | 103 | 14928.648 | 15448.004 | 0.777 |
| *Note*: N = 1144. All values represent the mean across 20 imputed datasets. Some LCA model fit statistics not presented with imputed data. LL = Log-likelihood statistic;  AIC = Akaike information criterion; BIC = Bayesian information criterion;  Relative entropy is a summary measure of classification certainty once posterior class  probabilities are obtained and can be computed for k > 1-class models. | | | | | |
